# Understanding underperformance in a high-stakes clinical-based simulation assessment in physiotherapy: a descriptive analysis

**DOI:** 10.1186/s12909-023-04649-8

**Published:** 2023-09-18

**Authors:** Brooke Flew, Belinda Judd, Belinda Lange, Darren Lee, Felicity Blackstock, Joanna Tai, Kathryn Tognon, Lucy Chipchase

**Affiliations:** 1 School of Allied Health, Human Services and Sport, La Trobe University, Melbourne, Australia; 2 Faculty of Medicine and Health, The University of Sydney, Sydney, Australia; 3 College of Nursing and Health Sciences, Flinders University, Adelaide, Australia; 4 Australian Physiotherapy Council, Melbourne, Australia; 5 School of Health Sciences, Western Sydney University, Sydney, Australia; 6 Centre for Research in Assessment and Digital Learning, Deakin University, Geelong, Australia

**Keywords:** Clinical reasoning, High-stakes assessment, Overseas qualified physiotherapists, Underperformance

## Abstract

**Background:**

High-stakes assessments are often used as a ‘gate-keeper’ activity for entry into the health professions by ensuring that the minimum core competency thresholds of the profession are met. The aim of the study was to explore if common areas of underperformance existed in international candidates assessed with a high-stakes clinical-based simulation assessment for entry into the physiotherapy profession in Australia.

**Methods:**

A retrospective mixed methods analysis of the clinical assessments completed by international candidates over a one-month period in 2021 that were deemed as not meeting competency. The clinical assessments were completed in one of the three practice areas: cardiorespiratory, musculoskeletal, or neurological rehabilitation. Each assessment was scored by two independent assessors, who discussed the performance and then completed a moderated assessment form. The assessment form used to score competency included seven domains such as initial assessment, effective treatment, communication skills, and risk management.

**Results:**

Fifty-one clinical assessments graded as not competent were analysed. Across the practice areas, a high failure rate was found in domains related to interpreting assessment findings and developing a treatment plan. This trend was also observed in the qualitative data, suggesting candidates struggled to meet competency in areas of planning and prioritisation, interpretation and implementation of the information gathered, and selection and evaluation of effective treatment.

**Conclusion:**

These findings align with published data on the underperformance of Australian physiotherapy students in clinical placement settings, suggesting these issues are not specific to high stakes assessment of overseas physiotherapists, and that education needs to focus on improving these skills within the profession at all levels. With the identified areas of underperformance aligning with the ability to use higher order thinking and skills integral to clinical reasoning, improvements in the education and implementation of clinical reasoning may be a place to start.

**Supplementary Information:**

The online version contains supplementary material available at 10.1186/s12909-023-04649-8.

## Introduction

All assessments, particularly high-stakes assessments, carry direct consequences for examinees, programs or institutions [1–3]. In health professional education, high-stakes assessments may prevent graduation or impact eligibility for admission, licensing or certification [4, 5]. High-stakes assessments are controversial since a single assessment may not accurately reflect the ability and knowledge of an examinee or be a holistic measure of competence [1, 2].

In health professional education, high-stakes assessments act as a ‘gate-keeper’ activity for entry into a profession. They ensure that the minimum core thresholds of the profession are met [6], highlighting the competencies required by accrediting agencies [7]. In the United States, overseas qualified physiotherapists must sit the National Physical Therapy Examination (NPTE), an assessment consisting of 250 multiple choice questions [8]. Similarly, until 2022, Canadian trained and overseas qualified physiotherapists seeking registration in Canada had to pass the Physiotherapy Competency Examination (PCE), consisting of written and clinical components [9]. High-stakes assessments of physiotherapists graduating from accredited programs are not conducted in Australia and New Zealand, however, overseas qualified physiotherapists whose qualifications are not deemed explicitly equivalent to Australian qualifications are required to sit high-stakes assessments in order to practice in Australia [10, 11].

In 2019, around 10% of Canadian qualified physiotherapy candidates failed the PCE, with 42.7% of overseas trained candidates failing [9]. These fail rates are similar in comparable licensing exams for other health professionals. For example, the fail rate for overseas qualified candidates in nursing was 54.5% but only 11.8% for American nurses in the American National Council Licensure Examination. For medicine, 23% of overseas qualified candidates failed the United States Medical Licensing Examination Stage 1 in 2021 compared to 5% of American/Canadian candidates [12, 13]. In the 2019–2020 financial year, approximately 50% of overseas qualified physiotherapists failed the Australian Physiotherapy Council’s (APC) high-stakes clinical-based simulation assessment.

Failure of overseas trained health professionals to demonstrate competency in licensing exams impacts the candidate, the administering entity, the workforce, and the diversity within the profession that arises from membership of global citizens. For candidates, the assessment process can be costly. Candidates may pay substantial fees, and each instance of failure represents a financial burden, which could contribute to increased stress levels and have negative implications for ease of settlement in the new country [14]. Stress and anxiety are purported to be major contributing factors to the high failure rates as they have been shown to impact working memory, retrieval of information, and decision making in health professionals [15, 16]. Stress during, and in the lead up to, high-stakes testing is common and has also been linked with a fear of not progressing and achieving career goals [17–20]. Indeed, if candidates fail to succeed in a high-stakes assessment, it may have deleterious effects, such as severe test anxiety, that may prevent success on future attempts [19]. Additionally, the theory behind why individuals experience stress suggests that it is related to their perception of demand versus resources [15, 21]. That is, we experience stress only when we perceive the demand of a task is greater than our resources, such as skill level and experience, to address it. If our resources are adequate to meet the demand of a task, then it is perceived as a challenge instead of a stress. As such, understanding the areas of an assessment in which candidates may struggle could provide insights for initiatives that aim towards improving their resources in these areas, potentially resulting in a reduced likelihood of a stress response and improved performance.

With these considerations around high-stakes assessment, it is important to understand the patterns of examinee underperformance (i.e., not meeting the competency standard threshold). There is limited documented research regarding patterns of failure of high-stakes assessment from the perspective of candidates undertaking overseas licensing exams and examiners. Research into the performance of health professional students (pre-registration) on clinical placements provides some insights. Common areas of failure include students demonstrating inadequate communication/reflection skills, lack of feedback literacy, poor goal setting skills, or issues interpreting assessment findings and selecting appropriate interventions [22–25]. However, these studies are in clinical placement settings with assessments completed over an extended period (longitudinal evaluations), allowing students to adapt and improve across the course of placement. High-stakes simulation one-off assessment outside the workplace may result in different patterns or aspects of inability to meet thresholds. For example, underperformance by overseas physiotherapists may reflect differences in the local expectations of physiotherapist performance or cultural differences in physiotherapy practice between countries. Understanding the areas of assessment where underperformance is observed may not only assist candidates to prepare more effectively but also highlight gaps for education providers, professional development providers, and the wider physiotherapy profession. Understanding these gaps highlights opportunity for development of initiatives that provide greater support to overseas trained physiotherapists in preparation for the examination. Thus, this study aimed to describe the trends observed with unsuccessful performance in a high-stakes clinical-based simulation assessment. Herein termed ‘clinical assessment’, these practical examinations determine eligibility of overseas qualified physiotherapists for registration to practice as a physiotherapist in Australia.

## Methods

### Design

A retrospective mixed methods analysis of internationally trained physiotherapists’ performance observed in the clinical assessment was conducted. The clinical assessment is current practice for the APC and involves candidates assessing and managing standardised patients across three areas of physiotherapy practice: musculoskeletal, neurological, and cardiorespiratory practice. A de-identified APC data set of assessment performance and outcomes was extracted for analysis. Only clinical assessments considered to have “not met competency” and completed in a one-month period in 2021, were used in the analysis.

### Context and assessment

In Australia, overseas qualified physiotherapists must pass an eligibility check for suitability for registration with the Physiotherapy Board of Australia. If their entry-level training is not deemed equivalent to an accredited Australian entry-level program, candidates need to sit both a theoretical and clinical assessment. The theoretical component consists of a written examination. Once passed, this is followed by the clinical assessment, being three face to face simulation-based assessments (one in each practice area) [10, 26]. The APC’s decision to use simulation-style assessment was in response to the move in health profession education where simulation was being used as a tool for education and assessment [27–31]. This was also in response to difficulty accessing public hospitals for assessment purposes and large assessment wait times [26].

Each patient case scenario in the clinical assessment is developed to closely replicate an authentic clinical presentation with a real patient. The candidate is provided approximately 75 min in the simulation-based assessment to demonstrate meeting the thresholds of practice indicated on the assessment form. Every candidate is assessed concurrently by two independent assessors, one being a specialist in the area being examined and the other a generalist physiotherapy practitioner. Assessors are provided with a comprehensive assessor’s manual and are trained by the APC on the use of the assessment form to ensure consistency of marking. Patient actors are trained by the APC using standardised case scenarios and training protocols to portray patient roles, as is commonly conducted in health professional education and assessment [32, 33]. All clinical assessments are conducted in person at the APC simulation lab in Melbourne, Australia. Candidates must successfully complete all three clinical assessments to be licensed to practice in Australia (Further details at: https://physiocouncil.com.au/overseas-practitioners/standard-assessment-pathway/clinical-assessment/).

### Assessment form

Performance of competency in the clinical assessment is scored on an assessment form containing six domains (Additional file 1). The six domains include: 1 A) collect patient information and form a preliminary hypothesis, 1B) design and conduct a safe assessment, 2) interpret and analyze the assessment findings, 3) develop a physiotherapy intervention plan, 4) implement safe and effective physiotherapy interventions, 5) evaluate the effectiveness and efficiency of physiotherapy intervention(s) and 6) communicate effectively. Each domain has between 2 and 6 sub items, which provide further details on components of the domain. There are also two global pass/fail ratings: 7) risk management incidents and 8) overall performance. All domains and global ratings are scored as either ‘yes’ or ‘no’ translating to competent or not competent respectively. To pass each clinical assessment, all domains and global ratings must be scored as competent.

The domains in the assessment form are based on the Physiotherapy Practice Thresholds in Australia and Aotearoa New Zealand [34]. The Physiotherapy Practice Thresholds were created as indicators of the level of competence that new and continuing physiotherapists in Australia must demonstrate for initial and ongoing registration to practise.

### Data management and analysis

#### Descriptive analysis

The data were exported from the APC database into Excel and converted to numerical data. The data set was then cleaned to check for missing or incorrectly entered data. A descriptive analysis of Domains 1 to 7 was completed, with the final global rating domain 8 excluded. Domain 8 was excluded from further analysis as it reflects the outcome of the assessment and as we selected only assessments marked as not competent, there was no need to analyse this domain. The descriptive statistics reflected the percentage of assessments determined not competent in each domain. The data were entered into SPSS (version 28), a Pearson’s Chi Square was then used to determine whether there was a statistically significant difference in the proportion (percentage) deemed not competent across the three areas of practice. Statistical significance was set as p ≤ 0.05.

#### Written comments

During the clinical assessment, written comments from assessors are recorded for each domain within the assessment form. These written comments were downloaded from the de-identified database and reviewed as complementary qualitative data. Not all clinical assessments had comments recorded as comments are optional. Comments from the assessment forms were managed in Microsoft Word software at all stages of the analysis.

Assessor comments underwent an inductive thematic analysis at the semantic level [35, 36]. An inductive approach was required due to the under-researched nature of this study. Inductive thematic analysis allows meaning and knowledge to be constructed that is data driven without the use of pre-existing codes [37]. The six phases of thematic analysis were used when coding being initial familiarization of the data by BF, initial coding of data by BF, generating themes from codes completed by BF and LC, reviewing and refining these themes against the dataset by BF, defining and naming themes completed by the whole research team, and writing up themes completed by the whole research team. To ensure credibility, transferability, criticality, and confirmability of the findings, several strategies were used [38]. As the data was collected during usual practice and through written comments, an audit trail was not needed for this process. Regular peer debriefs occurred through the data analysis stage with the whole research team to ensure critical analyses of findings. Personal assumptions and biases of the researchers were challenged and acknowledged. At the time of data analysis, BF had minimal previous experience with overseas qualified physiotherapists and the assessment process but is a qualified physiotherapist practicing in Australia. LC is an academic staff in physiotherapy, an experienced researcher in physiotherapy education and is associated with the APC. Themes were re-examined, re-interpreted, and refined during data analysis. A reflexive journal was also kept during data analysis by BF to increase the rigour of the findings [39].

## Results

A total of 51 clinical assessments achieved a ‘fail’ as competency was not demonstrated in one or more of the domains or global ratings. This resulted in 102 assessment forms being completed (two per assessment) across a sample of 31 candidates who failed one (n = 16), two (n = 10), or three (n = 5) of the clinical assessments. The assessments were in cardiorespiratory (n = 34, 33%), musculoskeletal (n = 30, 30%), and neurological physiotherapy (n = 38, 37%) areas of practice. Further demographic detail was withheld to ensure anonymity of candidates.

### Failure rates across the domains and practice areas

When all practice areas of the clinical assessments were grouped together, there was a high failure rate overall in domains 1B to 5 (77.5–87.3%), with only domains 6 (29.4%) and 7 (33.3%) having less than 50% of assessments deemed “not competent” (Table 1). Individually, musculoskeletal assessments were not performed well in domain 2 (80%) but performed very well in domain 7 (6.7%). Cardiorespiratory assessments were not performed well in domain 4 (91.2%) but very well in domain 6 (38.2%). Finally, neurological physiotherapy assessments were not performed well in domain 4 (100%) but very well in domain 6 (36.8%). The chi-square analysis demonstrated no significant difference in fail rates for domains 2 and 3. This demonstrates that the fail rates were consistent across practice areas. Considering the percentages by physiotherapy practice area, neurological cases attracted the highest percentage of candidates scored as not competent.

**Table 1:** The percentage (%) of assessments rated as failure in each domain for the three clinical areas

Domains	Failure percentage				Pearson’s Chi-Square	Asymptotic Significance
	MSK (n = 30)	Cardio (n = 34)	Neuro (n = 38)	Overall (n = 102)		
1 A Collect Patient Information and Form a Preliminary Hypothesis	43.3	41.2	71.1	52.8	8.00	0.02
1B Design and Conduct a Safe Assessment	70	67.7	92.1	77.5	7.50	0.02
2 Interpret and Analyse the Assessment Findings	80	82.4	89.5	84.3	1.29	0.53
3 Develop a Physiotherapy Intervention Plan	70	70.6	89.5	77.5	5.02	0.08
4 Implement Safe and Effective Physiotherapy Interventions	66.7	91.2	100	87.3	17.46	< 0.001+
5 Evaluate the Effectiveness and Efficiency of Physiotherapy Intervention(s)	60	81.8	94.7	80.2	12.82	0.002
6 Communicate Effectively	10	38.2	36.8	29.4	7.73	0.02
7 Risk Management Incidents	6.7	50	39.5	33.3	14.49	< 0.001

*Abbreviations: MSK = musculoskeletal, Cardio = cardiorespiratory, Neuro = Neurological rehabilitation*

### Written comments

From the 102 ‘failed’ clinical assessment forms, a total of 285 written assessor comments were provided, with comments received across all domains. The percentage of comments varied depending on domain from 12% of assessment forms to 45% of assessment forms. Comments were only made with respect to domains not performed well by candidates, as such there were no comments indicating areas performed well and no deviant opinions requiring further analysis or acknowledgement.

Many comments understandably aligned with the areas of higher failure rates highlighted in the descriptive data analysis. Overarchingly, there were three themes from the analysis of the qualitative data. The first theme related to candidates and their consideration of patients’ values, needs, and goals. The second theme relates to the candidate’s own processing of information. The third theme relates to the patient’s outcome. These themes are discussed below, and quotes included to support the themes and illustrate the findings.

#### Two Ps- planning and prioritizing

Assessors consistently reported a feeling that candidates were not adjusting their plan and treatment based on the patient presentation and priorities. There was a feeling that candidates were using a rote-learning approach and implementing a pre-prepared plan that did not consider the specifics of the patient of their assessment findings. This is evidenced by the following:

*Treatment seemed quite recipe based and was not developed with a clear understanding of patient’s deficits or goals.*

*Did not prioritise assessments [sic] plans according to patient presenting symptoms and anxiety levels.*

Some comments also suggested candidates struggled with or omitted discussion of goals completely, meaning they were unable to use these to guide their plan for the session to ensure it was aligned with the patient’s priorities:

*Did set goals with patient but intervention did not address what patient wanted which was to be able to do more with right shoulder*.

*No priorities or goals discussed with the patient at any time. No sense of patient priorities.*

#### Two Is- interpreting and implementing

This theme is a logical progression from the first, where candidates, having completed some level of planning and prioritising of the specific patient, are then required to interpret and implement this information. First, candidates appeared to struggle with the ability to appropriately analyse and interpret the assessment. Second, they struggled to use the findings to guide their decision making and clinical reasoning. Often missing key information or improperly using information to justify a decision, hence, being unable to correctly implement their findings to guide an effective treatment plan. This seemed to stem from either inadequate clinical reasoning skills or inadequate information collection in the assessment, leading candidates to have limited information to draw upon when attempting to justify choices. The following quotes highlight this:

*Clinical reasoning unclear and did not make links between the assessment and treatment undertaken.*

*Because objective assessment was lacking, could not develop a logical rationale for physiotherapy intervention, nor select appropriate and effective interventions.*

#### Two Ts- treatment and testing

The final theme relates to the choices that candidates made around interventions for the patient and their ability to know if this intervention was effective. Assessors described issues with candidates’ abilities to choose treatments that would be effective. This appeared to relate to treatments not being related to the patient presentation, as discussed in the first theme, or they were not able to adequately progress an intervention to be appropriately challenging for the patient. Related was the candidate’s ability to re-assess and evaluate the effectiveness of their treatment. As many candidates appeared not to re-assess or selected the wrong assessments, it was not possible for them to have the information to justify if their treatment was appropriate and effective for the specific patient:

*Did not observe/correct patient during treatment, did no[t] reassess objectively, did modify intervention when done poorly or when patient not sure what they were doing*.

*Attempted to reassess some outcome measures but these were not appropriate given her treatment focus*.

## Discussion

This is the first published study to report areas of underperformance in a high-stakes clinical-based simulation assessment of overseas trained physiotherapists seeking entry to the Australian physiotherapy profession. Across the three areas of clinical assessment, domains pertaining to collecting information, communication, and risk management were generally performed well by unsuccessful candidates, with 52.8%, 29.4% and 33.3% failure rates respectively. However, all other domains, capturing the skills of assessment, planning, implementing interventions, and evaluating, had a 77.5% or greater failure rate across the three areas of practice, although there was variability in the performance of domains across the areas. However, the ability to interpret and analyse assessment findings and thereby develop a physiotherapy plan (domains 2 and 3) were consistently underperformed in all three areas of practice, highlighting that these are key areas that potentially underpin failure to succeed. These areas of underperformance were corroborated by the written comments that had themes around issues with planning and prioritising for the specific patient, interpreting and implementing the information gathered, and selecting effective treatments and assessing for effectiveness.

Bloom’s revised taxonomy levels of cognitive learning supports the findings of this study in that the domains performed well by candidates, those involving collecting information and communicating effectively (Domains 1 A and 6), are linked to less complex levels of remember and understand [40]. The areas that were not commonly met (interpreting, analysing, and implementing) unsurprisingly, required the higher levels of cognitive learning from candidates. Domains 2 through to 5, which had higher overall rates of failure, were those that required complex levels of learning, including analysis, evaluation, and creation [40]. These domains and associated more complex cognitive learning processes are related to a candidate’s ability to demonstrate sound clinical reasoning that involves the thinking and decision-making processes associated with clinical practice [41, 42]. A candidate’s ability to use complex levels of learning to clinically reason underpins all domains assessed in the APC clinical assessment but is key to the domains that candidates found most challenging, such as interpret and analyse findings, implement an effective treatment, and evaluate effectiveness. The link to clinical reasoning skills was triangulated with the written feedback that identified issues such as selection of ineffective treatments, an inability to modify for the specific patient presentation, and a limited ability to reassess or re-evaluate. This suggests that while candidates were challenged across a range of domains, the underlying factor in their result was their ability to demonstrate and apply sound clinical reasoning to individual patient cases.

These findings are similar to those reported by Dalton, Davidson and Keating (2011) who examined the results of 456 Australian and New Zealand physiotherapy students on clinical placement. In this study, the most common areas of underperformance were the limited ability to set goals, progress an intervention, and interpret assessment findings [23]. Similarly, Judd et al. (2016) reported the performance of 1260 Australian physiotherapy students completing a placement in clinical and simulation settings. Areas in which students failed to perform competently included their ability to select and measure relevant health indicators and outcomes, set realistic short- and long-term goals, appropriately interpret assessment findings, and perform appropriate intervention [25]. These studies evaluated the performance of physiotherapy students during their clinical placement and in a simulated environment. While it is acknowledged that the student population is different from overseas qualified physiotherapist with potentially years of practice experience, high-stakes assessments of physiotherapy clinical competence appear to have commonalities in terms of the domains where competency is harder to reach. Put plainly, these results suggest that regardless of country of training, progress through training (undergraduate/postgraduate), and assessment method utilised, the most challenging areas in which to reach competency are the ability to set relevant goals, interpret assessment findings, and create effective treatment plans, and that there is an interplay with these and the ability to clinically reason and use higher-level thinking.

The results also highlighted differences in performance across areas of clinical practice. Domains 2 and 3, interpret and analyse findings and develop an intervention plan, were consistent across all areas of practice, again supporting the idea that the skills of higher-level thinking and clinical reasoning are key to physiotherapy practice. In the area of neurological practice, candidates had significantly higher levels of underperformance in the first two domains, collect information and conduct an assessment, compared to musculoskeletal and cardiorespiratory practice. Assessments in musculoskeletal practice on the other hand had lower levels of underperformance in the last four domains, implement intervention, evaluate effectiveness, communication, and risk management. No previous data of similar high stakes physiotherapy assessments have been reported, so it is difficult to interpret and compare these findings. However, Australian students completing placements were found to have better scores in the area of musculoskeletal practice compared to cardiorespiratory and neurological practice [43]. This difference may be due to more rigid frameworks and clinical patterns involved in musculoskeletal assessment and treatment. Further research into the effect of area of practice on candidate performance and its impact on education and assessment may be beneficial.

Overall, this study found common areas of underperformance by overseas qualified physiotherapists in high-stakes clinical-based simulation assessments. This information can be used by educational institutions to aid the construction of targeted training, learning, and professional development opportunities to support internationally credentialled physiotherapists immigrating to Australia or countries with equivalent entry level training, such as the United States and Canada. The results may also be used to provide feedback to potential or previously unsuccessful candidates and entry level physiotherapists around areas that they may need to focus on to reach competency levels expected by the profession. More research is recommended into the common areas of underperformance (planning and prioritising for the specific patient, interpreting and implementing the information gathered, and selecting effective treatments and evaluating their effectiveness) to investigate if similar results would be found internationally. Additionally, research into fostering clinical reasoning and the acquisition of skills needed in the identified areas of weakness, may help shape the future training of physiotherapists to bridge any such gaps.

### Limitations

This study was descriptive in nature, such that the results need to be considered carefully when attempting to generalise the findings to other populations and cannot be used to determine causality. Despite this, the findings align with previous research in other cohorts and contexts, strengthening our understanding of the areas that physiotherapists find challenging when demonstrating competency and trying to reach thresholds for practice. Due to the small sample size, this data may not be representative of the full breadth of overseas qualified physiotherapists. However, the percentage of exams failed in each area of practice, the failure percentage across the domains, and the number of candidates failing one, two, or three exams is consistent with the percentages observed in the entire cohort for the 2020–2021 financial year. This suggests our sample was representative of the typical candidate applying for assessment for suitability for Australian registration through the Standard Assessment Pathway.

## Conclusion

This study identified that there are common factors in underperformance in a high-stakes clinical-based simulation assessment for international physiotherapists applying to practice in Australia. Clinical reasoning, including its relationship to selecting contextually appropriate patient treatment and assessment, is a substantial area for development, and is highly critical for safe and effective professional practice. Additional studies investigating the areas of underperformance within physiotherapy and why clinical reasoning skills may vary for international candidates are needed.

### Electronic supplementary material

Below is the link to the electronic supplementary material.

Supplementary Material 1

## Data Availability

The data that support the findings of this study are available from the APC but restrictions apply to the availability of these data, which were used under license for the current study, and so are not publicly available. Data are however available from the authors upon reasonable request and with permission of the APC.

## List of Abbreviations

APC: Australian Physiotherapy Council
Cardio: Cardiorespiratory
MSK: Musculoskeletal
NPTE: National Physical Therapy Examination
Neuro: Neurological rehabilitation
PCE: Physiotherapy Competency Examination
