# Supplementary figures and images for: Understanding underperformance in a high-stakes clinical-based simulation assessment in physiotherapy: a descriptive analysis
Source: BMC Med Educ. 2023 Sep 18;23:676. doi: 10.1186/s12909-023-04649-8 (PMC10506262; doi:10.1186/s12909-023-04649-8)

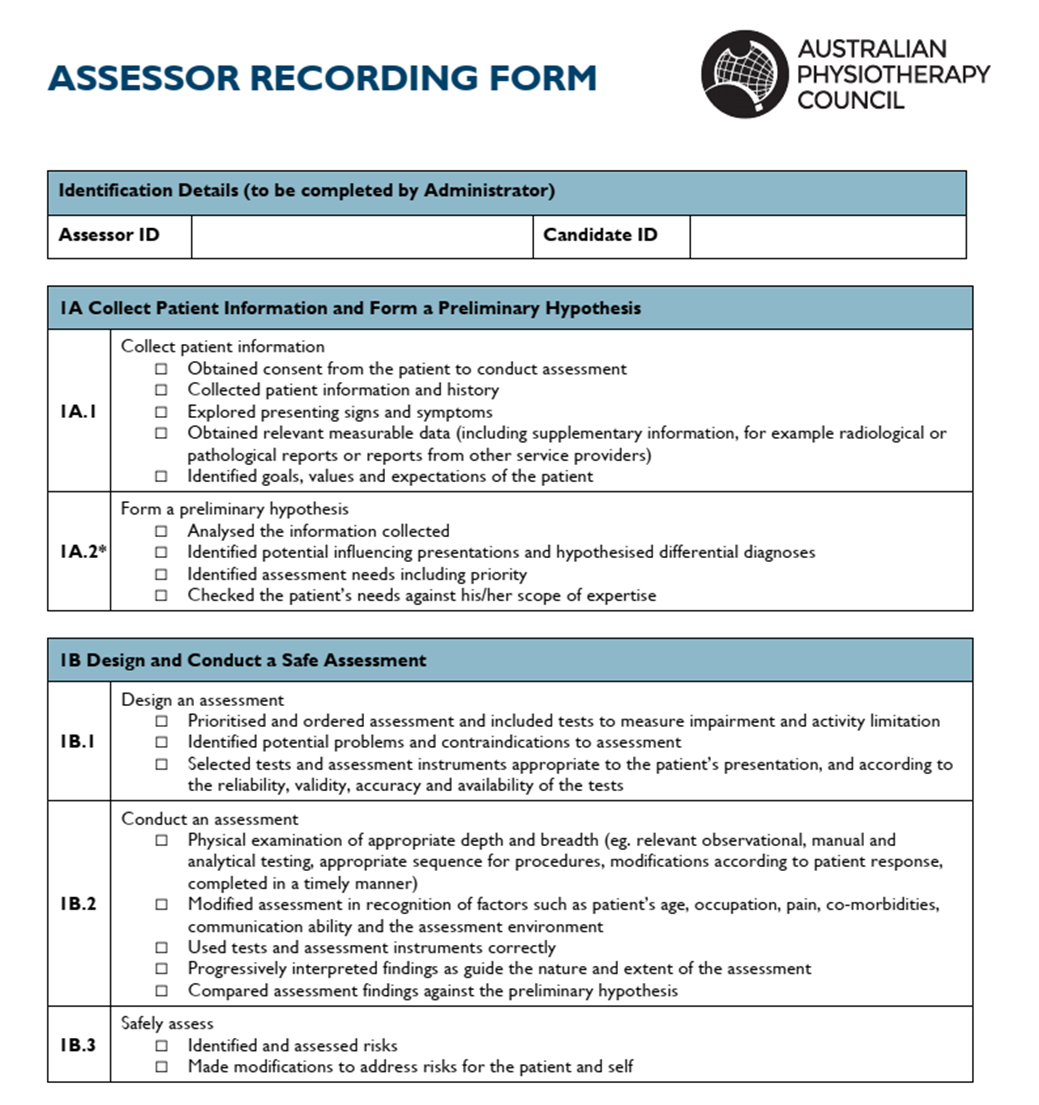


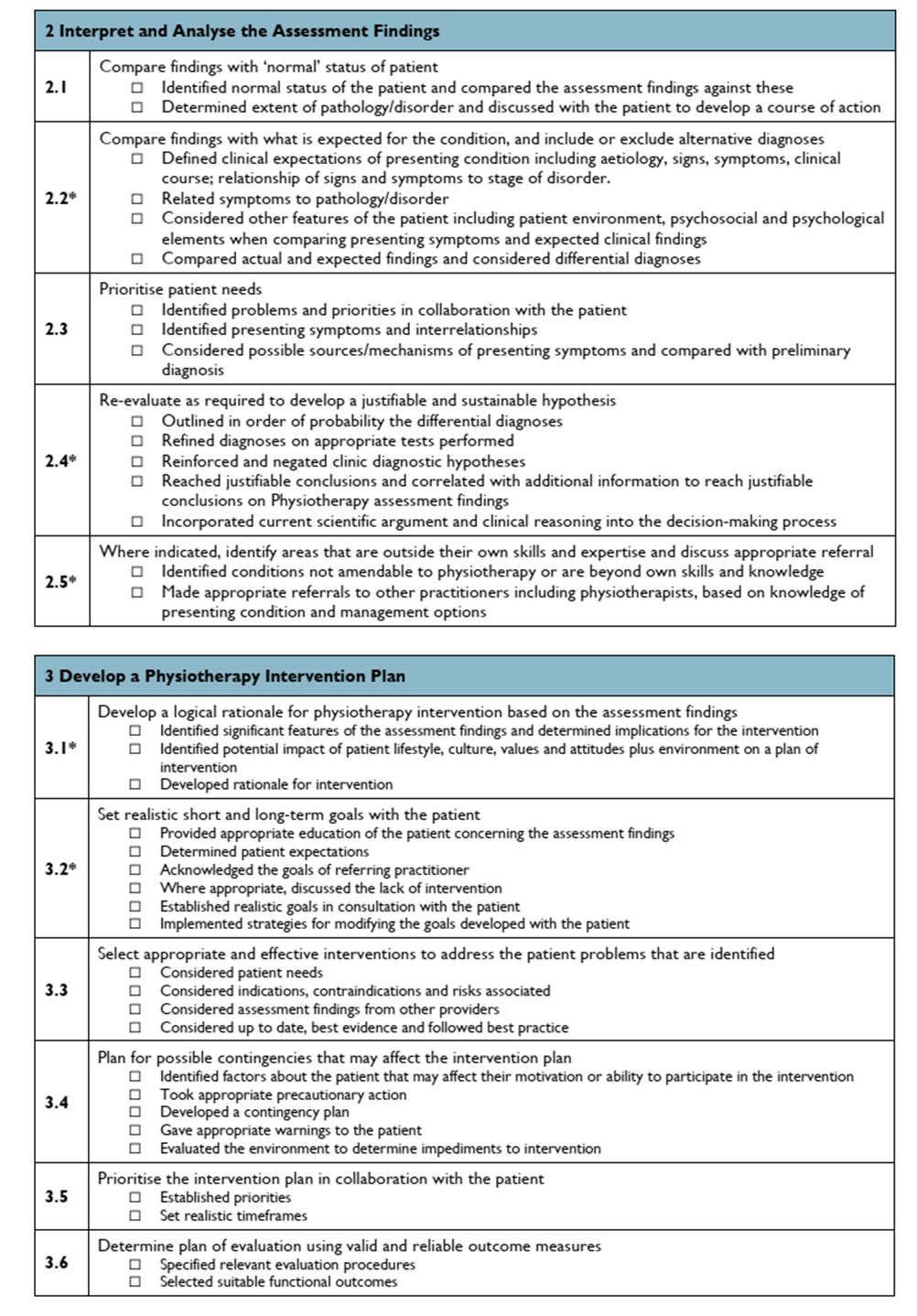


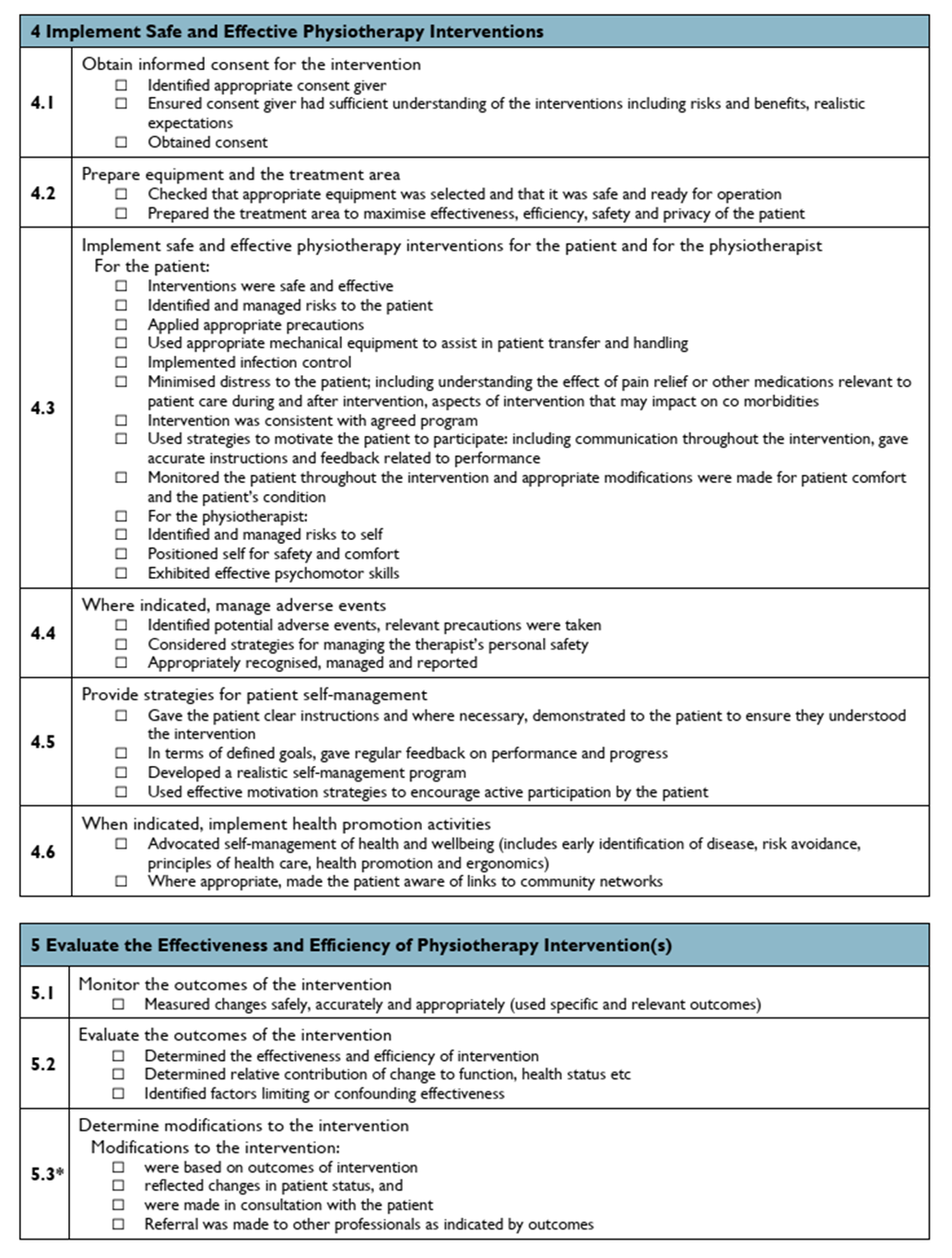


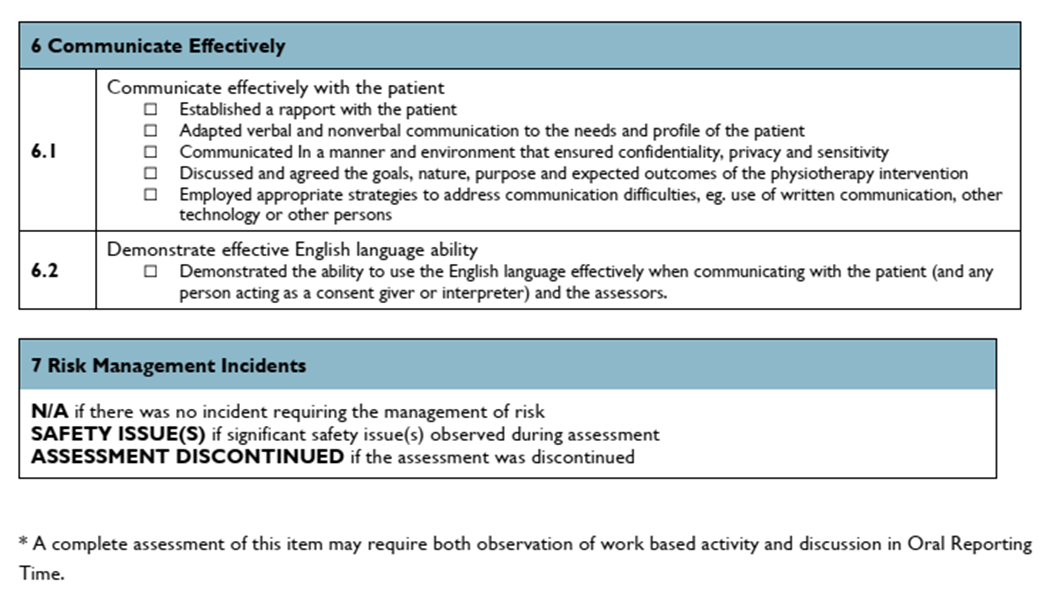

Supplement: Supplementary file 1 — Supplementary Material 1 [file 12909_2023_4649_MOESM1_ESM.docx]
